# Clinical characteristics and biomarkers feature analysis using a proteomics platform in young patients with acute coronary syndrome

**DOI:** 10.3389/fcvm.2024.1384546

**Published:** 2024-08-13

**Authors:** Kandi Zhang, Fengdan Wang, Quan Yu, Yanqiong Song, Jun Gu, Qing He, Junfeng Zhang

**Affiliations:** Department of Cardiology, Shanghai Ninth People’s Hospital, Shanghai Jiaotong University School of Medicine, Shanghai, China

**Keywords:** acute coronary syndrome, proteomics, premature coronary artery disease, osteopontin, myoglobin, growth differentiation factor 15

## Abstract

**Background:**

Acute coronary syndrome (ACS) is a leading cause of morbidity and mortality worldwide. In recent years, ACS has been reported to be associated with age, and the incidence has become more common in younger patients. Previous studies have identified various risk factors that contribute to the stratification of ACS patients. However, it remains unclear whether these risk factors, along with proteomic and clinical characteristics, are applicable to young ACS patients, as they are for middle-aged and elderly patients. This study aimed to investigate the proteomics, risk factors, and clinical characteristics of young ACS patients, as well as the differences between them and middle-aged and elderly ACS patients. By comparing these findings with those of middle-aged and elderly patients, we aimed to identify any discrepancies and these findings possibly may have implications for future management strategies of this specific population.

**Methods:**

This observational study included a total of 187 participants diagnosed with ACS and 17 young healthy individuals as the control group. ACS patients were divided into three age groups: <45 years old, 45–60 years old, and 61–75 years old. The control group consisted of healthy individuals under the age of 45 who underwent coronary angiography and were excluded from CAD. We collected clinical characteristics, laboratory data, and echocardiographic results from each participant. Additionally, blood samples were collected for further analysis of relevant proteomic and arteriosclerosis marker data using proteomics analysis.

**Results:**

Our findings revealed that the presence of certain key factors was associated with a significantly difference in patients with ACS aged younger than 45 years, and this association differed from that of traditional cardiovascular risk factors in patients older than 45 years. Specifically, a higher body mass index and hyperlipidemia were found to be associated with an increased risk of ACS morbidity in young adults (<45 years old) compared to middle-aged and elderly individuals. Furthermore, our findings indicated that the expression levels of growth differentiation factor 15, osteopontin, and NT-proBNP were significantly different among the groups.

**Conclusion:**

In summary, our study revealed that the main pathogenic factors of ACS patients under 45 years of age differed from those of middle-aged and elderly patients. These findings may contribute to the prevention and treatment strategies for young patients with ACS.

## Introduction

Despite significant advancements in the evidence-based management of acute coronary syndrome (ACS), it continues to cause high levels of morbidity and mortality worldwide (1). ACS affects a substantial portion of the younger population, accounting for approximately 20%–30% of cases, and remains a leading cause of death in both developed and developing countries. According to the Global Registry of Acute Coronary Events (GRACE), patients with ACS have a mortality rate of approximately 15% after one year and a cumulative mortality rate of up to 20% after five years (2, 3). Therefore, studying the risk factors for young ACS patients and implementing appropriate interventions are key focuses for reducing the mortality rate in this population.

The impacts of obesity, diabetes, hypertension, hypercholesterolemia, smoking, and a sedentary lifestyle on subsequent cardiovascular events have been well established as standard modifiable cardiovascular risk factors (SmuRFs) (4). Age has also been identified as a significant factor in ACS (5). However, due to changes in lifestyle, poor diet, and social pressures, there has been an increasing trend of coronary artery disease (CAD) among younger individuals (6). Based on the physiological and psychological characteristics of modern individuals, the World Health Organization classifies individuals aged less than 44 years as young, those aged 45–59 years as middle-aged, and those aged more than 60 years as elderly (7–9). Premature coronary artery disease (PCAD) and acute myocardial infarction (AMI) are terms used to describe CAD and AMI in young patients, with the definition of “young” varying in different published reports, ranging from 45 to even 40 years old. Several researchers estimate that approximately 2%–10% of all AMI patients are affected by morbidity among young adults (10).

Previous studies have emphasized the significance of various factors in risk stratification for recurrent cardiovascular events in ACS patients (11). Proteomics is one of the most promising fields in molecular biology, with tasks including the study of gene expression protein products, including their post-translational modifications and comparative analysis (12, 13). Proteomics research is a systematic study of all proteins in the body, studying the role of each protein in various physiological and pathological processes, and determining the potential use of proteins as effective diagnostic markers (14). In recent years, mass spectrometry analysis has been gradually introduced into ACS disease models and has made some progress in this filed. The PEA (Proximity Extension Assay) experimental method has played an important role in proteomics research due to its advantages of high flux, high sensitivity, high specificity, wide dynamic range, few sample requirements, targeting, and data stability, providing strong technical support for researchers (15). However, it remains unclear whether these risk factors with proteomic and clinical characteristics, are applicable to young ACS patients in contemporary management and prognosis of early-onset coronary heart disease (16). Additionally, even though recent relevant literatures have summarized disease management strategies, there is a lack of well-established guidelines or consensuses on the prevention and treatment of early-onset coronary heart disease (17, 18). Therefore, the objective of our study was to investigate whether the proteomics, risk factors, and clinical characteristics of young ACS patients under 45 years of age differ from those of middle-aged and elderly ACS patients. By analyzing data from a retrospective observational cohort, we aimed to provide valuable insights into effective predicting and prevention strategies for young ACS patients.

## Materials and methods

### Study population

**Figure 1:**
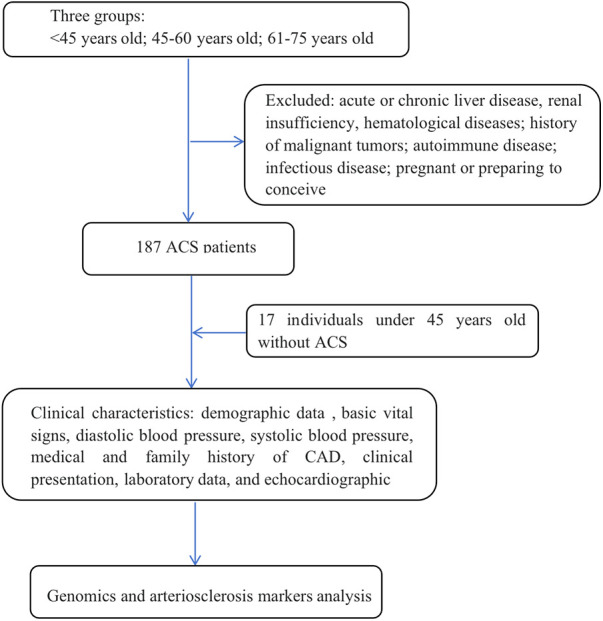
Flowchart of the selection process and data assessments of the present study.

This retrospective observational cohort study aimed to investigate the characteristics of patients with ACS across different age groups. The study included patients aged 18–75 years who underwent coronary angiography (CAG) at Shanghai Ninth People's Hospital Affiliated Shanghai Jiao Tong University School of Medicine between January 2021 and December 2021. The definition of ACS included non-ST-segment elevation myocardial infarction (NSTEMI), ST-segment elevation myocardial infarction (STEMI) or unstable angina pectoris. Patients with acute or chronic liver disease, renal insufficiency, hematological diseases, a history of malignant tumors with a life expectancy of less than 2 years, autoimmune disease or long-term immunosuppressant treatment, or infectious disease or who were pregnant or planning to conceive were excluded. Ultimately, a total of 187 ACS patients were included in the analysis (Figure 1). The ACS patients was divided into three age groups: <45 years, 45–60 years, and 61–75 years. Additionally, a control group consisting of individuals under 45 years old without confirmed CAD was included as control group compared to ACS patients under 45 years old, with a total of 17 patients.

Patient follow-up was conducted through outpatient clinical visits or telephone calls. The study was approved by the ethics committee of the Shanghai Ninth People's Hospital Affiliated Shanghai Jiao Tong University School of Medicine (Approval No. SH9H-2019-T160-6), and written informed consent was obtained. The study protocol adhered to the ethical guidelines of the 1975 Declaration of Helsinki.

### Data collection and assessments

All the data for this study were collected by three trained investigators from the electronic medical records system. To minimize bias, these investigators were blinded to the aim of the research. The collected clinical characteristics included demographic data (such as age, sex, occupation, economic income, marital status, and fertility status), basic vital signs [weight, height, body temperature, heart rate (HR), diastolic blood pressure (DBP), and systolic blood pressure (SBP)], medical and family history of CAD, clinical presentation, laboratory data, and echocardiographic results. Electronic medical records were used to gather this information. In addition to the clinical data, blood samples were collected from the participants who fasted overnight on the second after patient presentation. A total of 204 blood samples were collected and tested for various relevant proteomic information and markers of arteriosclerosis. These tests were also conducted to further analyze the genetic and biomarker profiles of the participants.

### Laboratory measurements

Plasma separation from blood was achieved through centrifugation. Whole blood was collected into commercial anticoagulant treatment collection tubes (treated with ethylenediaminetetraacetic acid). The tubes were then centrifuged in a refrigerated centrifuge at 1,000–2,000 × g for 10 min to separate the cells from the plasma. The resulting plasma was immediately transferred to clean tubes and stored at −80℃ until further analysis. For experimental detection, one microliter of plasma was used as the input material.

To analyze the plasma samples, we utilized the proximity extension assay (PEA) on the Olink platform. This method involves the use of validated antibody pairs that are linked to unique oligonucleotides. These antibody pairs bind specifically to their respective protein targets in the samples. The binding event generates a DNA amplicon, which is subsequently quantified using quantitative real-time polymerase chain reaction (PCR). The entire process consisted of an incubation phase, an extension and amplification phase, and a detection phase. During the incubation phase, the 92 antibody pairs, which were labeled with DNA oligonucleotides, bound to their target proteins in the samples. This incubation process typically takes approximately 16–22 h. In the extension and amplification phase, the hybridized oligonucleotides are extended using a DNA polymerase, resulting in the creation of a DNA barcode. This barcode was subsequently amplified viaPCR. The quantity of each DNA barcode was measured using microfluidic quantitative PCR (qPCR). Microfluidic qPCR was quantified using the Olink® Signature Q100, and the resulting data were analyzed using Olink® NPX Signature software. The incubation, extension, and detection steps of the experiment were performed by Sinotech Genomics Co., Ltd. (Shanghai, China).

To further analyze the obtained data, we conducted a Gene Ontology (GO) analysis for biological processes, cellular components, and molecular functions. Additionally, Kyoto Encyclopedia of Genes and Genomes (KEGG) pathway analysis and differential expression analysis was performed. These analyses were conducted using the Olink Analyze R package (version 3.1.0). Proteins with a *p* value less than 0.05 according to the t test were considered significantly differentially expressed and were retained for further analysis.

### Statistical analysis

Continuous variables are represented by the mean ± standard deviation (SD) or median (25th and 75th percentiles); an independent sample t test was used for continuous variable comparisons, and the Mann‒Whitney U test was used for nonnormally distributed variables. We used the Kolmogorov‒Smirnov test to test normality. Moreover, categorical variables are expressed as percentages and were evaluated using the chi-square test or Fisher's exact test. Nonparametric comparisons were used for comparing multiple groups with uneven variances. All the statistical analyses were conducted using IBM SPSS Statistics 25. *P* < 0.05 was considered to indicate statistical significance.

## Results

### Baseline characteristics

A total of 187 patients with ACS were included in the study. Among these patients, 37 were under 45 years old, 75 were between 45 and 60 years old, and 75 were between 61 and 75 years old. As a control group, a total of 17 individuals under the age of 45 without CAD. The baseline characteristics of the patients, as shown in Table 1, revealed several significant differences between the <45-year-old ACS group and the other age ACS groups. The incidence of hyperlipidemia was greater in the <45-year-old ACS group than in the other age ACS groups (*P* < 0.05). Additionally, compared with those in the other age groups, patients in the <45-year-old ACS group had a greater body mass index (BMI) (*P* < 0.05). In terms of family history of CAD, the prevalence was significantly greater in the <45-year-old ACS group (32.4%) than in the control group (*P *> 0.05). Similarly, smoking was found to be a significant factor among the various pathogenic factors, with a greater proportion of ACS patients having a smoking history than control group. However, there was no significant difference in smoking history among the three age groups. In terms of clinical presentation, there were no significant differences in HR, SBP, or DBP among the groups (*P *> 0.05).

**Table 1:** The baseline characteristics of the patients.

Clinical characteristics	<45 years old without ACS (control, *n* = 17)	<45 years old with ACS (*n* = 37)	45–60 years old with ACS (*n* = 75)	61–75 years old with ACS (*n* = 75)	P1	P2	P3
Male, *n* (%)	14 (82.4)	36 (97.3)	71 (94.7)	72 (96.0)			
Female, *n* (%)	3 (17.6)	1 (2.7)	4 (5.3)	3 (4.0)			
Age, year	39.12 ± 3.18	37.95 ± 3.75	52.23 ± 4.52	66.96 ± 3.95	0.000	0.000	0.270
BMI, kg/m^2^	24.09 ± 2.67	27.34 ± 2.48	25.36 ± 3.24	24.52 ± 2.84	0.000	0.000	0.000
Body temperature	36.42 ± 0.22	36.59 ± 0.22	36.54 ± 0.33	36.52 ± 0.23	0.158	0.193	0.007
Heart rate, bpm	83.29 ± 16.11	79.86 ± 11.26	76.79 ± 10.87	82.45 ± 15.97	0.054	0.921	0.370
Breathe	20.06 ± 7.43	19.00 ± 2.38	18.79 ± 1.47	19.17 ± 3.83	0.569	0.969	0.433
SBP, mmHg	132.35 ± 11.32	130.05 ± 19.92	130.45 ± 24.83	131.76 ± 24.59	0.970	0.810	0.593
DBP, mmHg	83.53 ± 8.49	80.81 ± 17.31	81.16 ± 15.29	78.49 ± 12.93	0.506	0.718	0.543
Family history of CAD, *n* (%)	4 (23.5)	12 (32.4)	29 (38.7)	23 (30.7)	0.583	0.798	0.506
Smoking history, *n* (%)	6 (35.3)	26 (70.3)	50 (66.7)	57 (76.0)	0.013	0.898	0.015
Drinking history, *n* (%)	3 (17.6)	16 (43.2)	23 (30.7)	29 (38.7)	0.219	0.331	0.067
History of hypertension, *n* (%)	5 (29.4)	17 (45.9)	38 (50.7)	46 (61.3)	0.083	0.272	0.251
History of diabetes, *n* (%)	1 (5.9)	9 (24.3)	13 (17.3)	20 (26.7)	0.201	0.762	0.105
History of hyperlipidemia, *n* (%)	2 (11.8)	5 (13.5)	6 (8.0)	6 (8.0)	0.753	0.296	0.859
K, mmol/L	3.85 ± 0.20	3.78 ± 0.29	3.82 ± 0.56	3.73 ± 0.46	0.638	0.971	0.368
Na, mmol/L	140.73 ± 1.75	138.56 ± 2.80	139.49 ± 2.87	137.25 ± 16.92	0.526	0.930	0.008
Cl, mmol/L	104.53 ± 1.60	102.67 ± 3.01	102.51 ± 11.14	101.64 ± 12.62	0.790	0.770	0.028
D-dimer, mg/L	0.15 ± 0.14	0.86 ± 2.88	0.40 ± 0.72	0.69 ± 0.81	0.256	0.560	0.368
PT, sec	10.95 ± 1.27	11.84 ± 2.69	12.77 ± 4.33	12.68 ± 6.15	0.488	0.372	0.132
APTT, sec	27.70 ± 3.61	37.33 ± 29.63	37.68 ± 23.45	42.19 ± 32.69	0.332	0.646	0.101
CRP, mg/L	1.89 ± 2.15	6.51 ± 16.81	8.32 ± 19.57	10.20 ± 16.77	0.380	0.426	0.315
cTnI, ng/ml	0.005 ± 0.01	14.20 ± 25.78	12.92 ± 24.95	11.17 ± 20.70	0.251	0.616	0.005
Mb, ng/ml	19.55 ± 7.11	451.65 ± 861.20	840.75 ± 1,187.13	709.04 ± 1,029.87	0.054	0.112	0.410
CK-MB, U/L	6.33 ± 7.42	61.82 ± 71.71	61.32 ± 89.20	74.06 ± 112.64	0.118	0.762	0.001
LDH, U/L	172.00 ± 33.86	267.68 ± 113.09	364.38 ± 469.01	301.05 ± 238.66	0.268	0.433	0.001
CK, U/L	106.79 ± 47.84	653.48 ± 729.29	653.26 ± 856.37	721.27 ± 1,110.47	0.163	0.859	0.001
HbA1c, %	5.76 ± 0.70	7.14 ± 2.38	6.38 ± 1.34	6.47 ± 1.80	0.062	0.110	0.004
AST, U/L	20.50 ± 5.16	86.65 ± 90.20	149.47 ± 255.49	93.35 ± 126.36	0.081	0.452	0.002
ALT, U/L	32.87 ± 25.11	50.63 ± 31.44	75.87 ± 168.59	33.61 ± 23.87	0.105	0.849	0.059
TC, mmol/L	4.49 ± 1.19	5.21 ± 1.48	4.62 ± 1.08	4.62 ± 0.96	0.040	0.030	0.101
TAG, mmol/L	1.81 ± 2.38	3.18 ± 3.25	1.82 ± 0.83	1.57 ± 0.89	0.000	0.011	0.147
HDL-C, mmol/L	1.12 ± 0.24	1.02 ± 0.36	0.95 ± 0.25	1.03 ± 0.27	0.105	0.634	0.316
LDL-C, mmol/L	2.95 ± 0.99	3.43 ± 0.98	3.25 ± 0.94	3.20 ± 0.76	0.336	0.202	0.127
LVEF, %	61.94 ± 3.94	56.73 ± 9.77	54.86 ± 8.34	53.36 ± 9.38	0.009	0.179	0.064

P1 (control vs. <45 vs. 45–60 vs. 61–75); P2 (<45 vs. 45–60 and 61–75); P3 (control vs. <45).

Regarding the laboratory data, the plasma low-density lipoprotein cholesterol (LDL-C) and high-density lipoprotein cholesterol (HDL-C) levels were similar among the groups (*P* > 0.05). However, among ACS patients, there were significant differences in total cholesterol (TC) and triglyceride (TAG) levels between the <45-year-old group and the elderly group (*P* < 0.05). Furthermore, the left ventricular ejection fraction (LVEF) significantly differed between ACS patients and healthy young individuals (*P* < 0.05), but there was no obvious difference in LVEF among the three age groups (*P* > 0.05).

### Differential protein and marker expression

**Figure 2:**
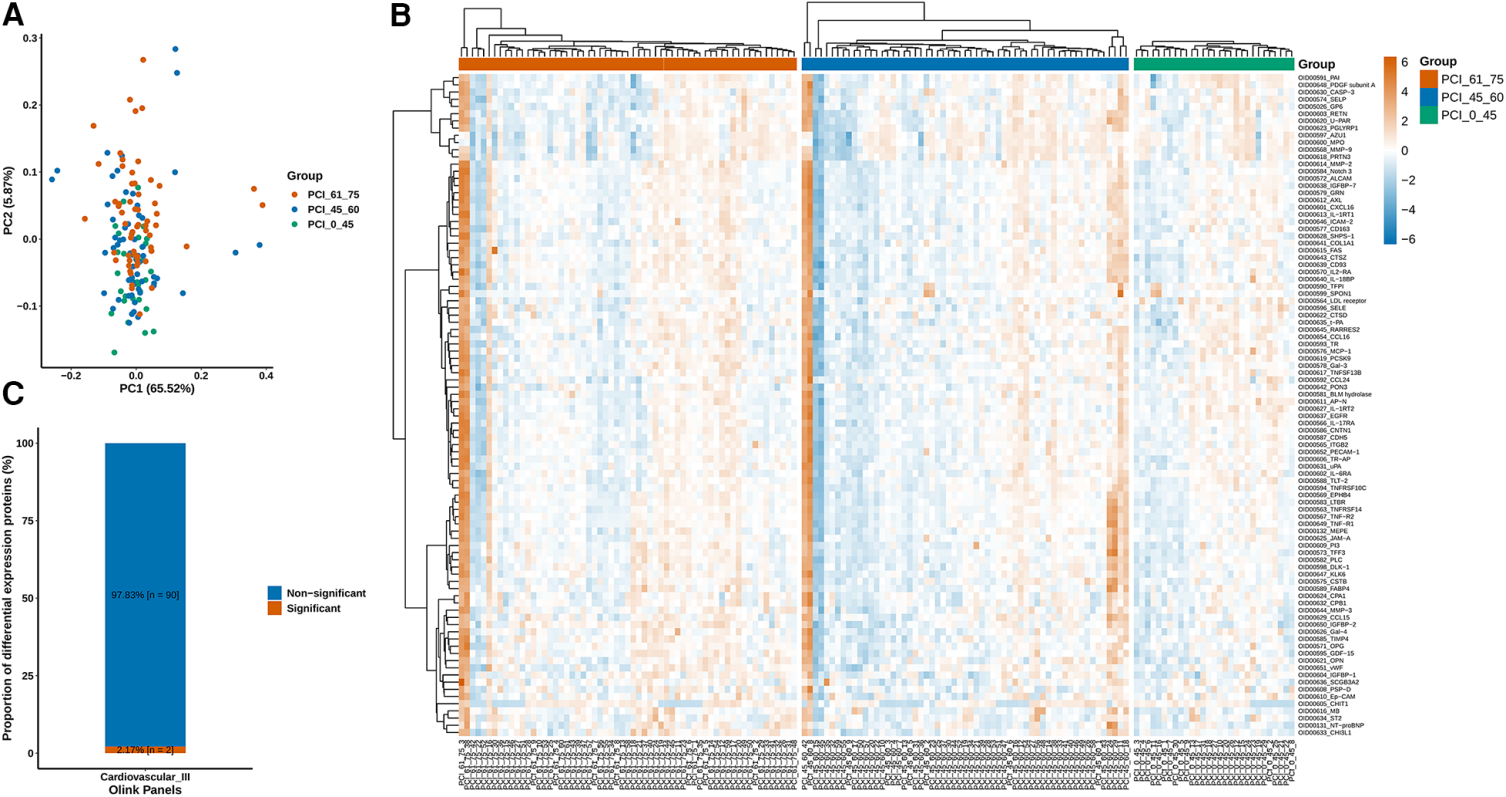
(**A**) According to the NPX value, the sample population had a smaller degree of dispersion in the <45-year-old group. (**B**) Diverse protein expression in the three groups was quantified. (**C**) The differentially expressed proteins accounted for 2.17% (*n* = 2) of the proteins, while the rest were nondifferentially expressed proteins.

**Figure 3:**
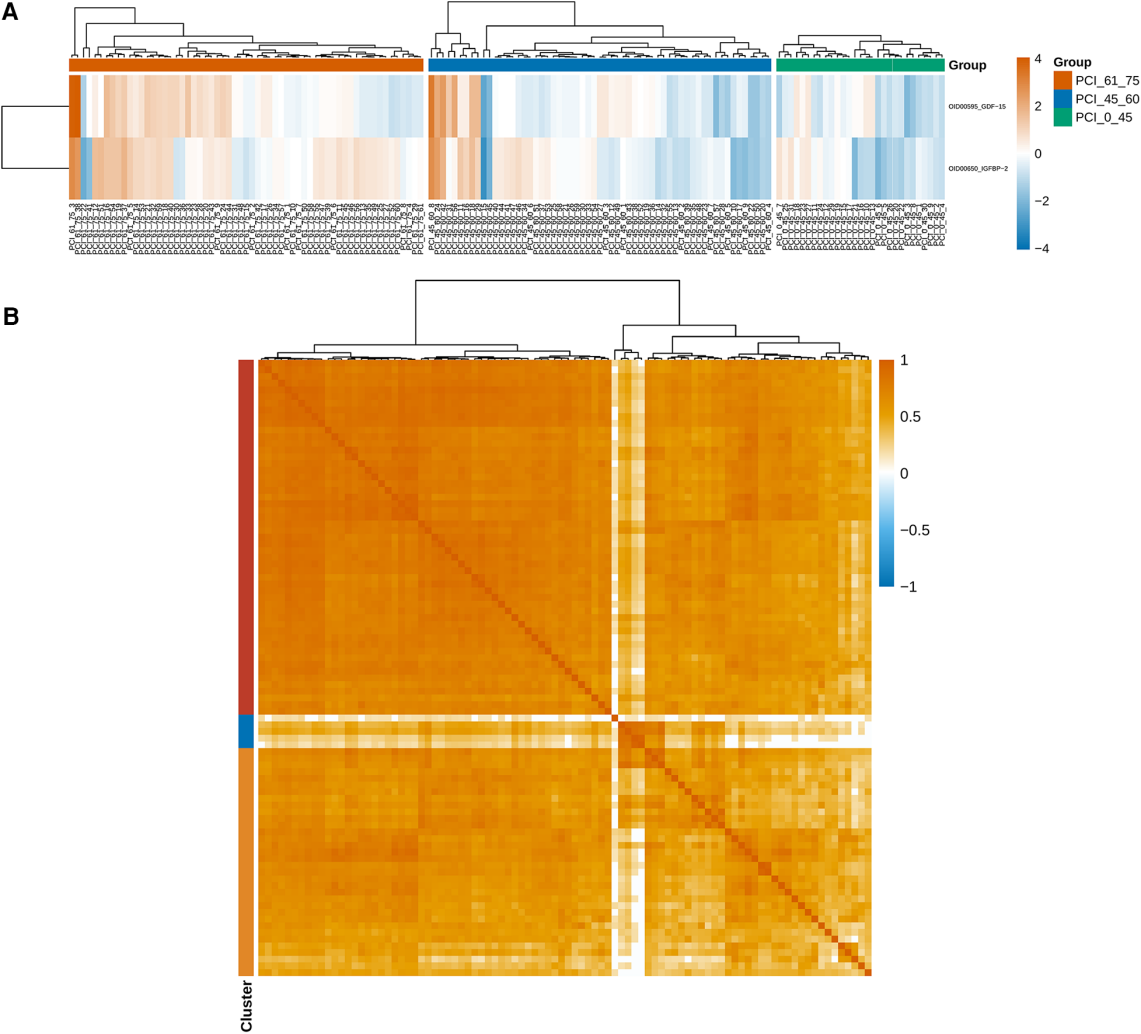
(**A**) The expression levels of GDF-15 and IGFBP-1 were significantly lower in young patients aged less than 45 years than in the other patients, and these findings are important for identifying ACS. (**B**) The Pearson correlation coefficient and protein–protein interactions were calculated.

The measurements were subjected to bioinformatics analysis. Supplementary Figure S1 displays the distribution of normalized protein expression in all samples to identify any outlier issues. Based on the NPX values, the <45-year-old group exhibited a smaller degree of dispersion than the other age groups (Figure 2A). The heatmap in Figure 2B shows the differences in protein expression among the three groups. Figure 2C indicates that only 2.17% (*n* = 2) of the proteins were differentially expressed, while the remaining proteins were nondifferential components. Specifically, the expression levels of growth differentiation factor 15 (GDF-15) and insulin-like growth factor binding protein 1 (IGFBP-1) in ACS patients aged younger than 45 years were significantly lower than those in the other groups, suggesting their potential importance in ACS (Figure 3A). Additionally, as shown in Figure 3B, the Pearson correlation coefficient and measured protein‒protein interactions were evaluated.

**Figure 4:**
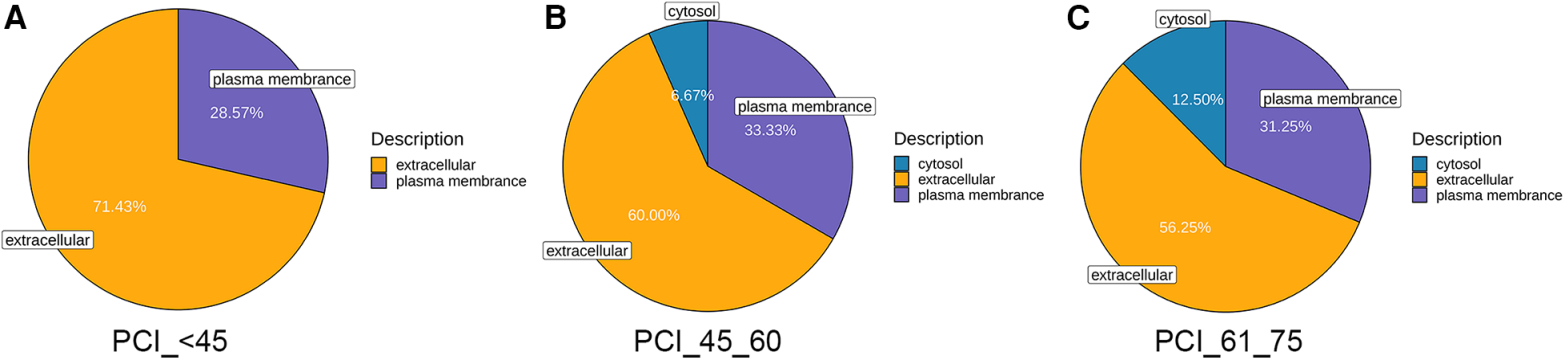
Mass spectrometry-based quantitative proteomic analysis of samples and classification of identified proteins. (**A**) Compared to those in normal blood samples, the differentially expressed proteins in samples from patients younger than 45 years were from the extracellular space and cytoplasm, followed by the plasma membrane. (**B**,**C**) The differentially expressed proteins in the samples from patients aged 45–60 and 61–75 years were primarily from the extracellular space and cytoplasm, followed by the plasma membrane and, ultimately, the cytosol.

The quantification of diverse proteins in the samples was also performed, and these proteins were classified into three categories based on GO annotation. As patients with ACS age, there is a gradual increase in the amount of proteins derived from the cytosol, whereas the proteins originating from extracellular sources decrease. In contrast, the levels of proteins from the plasma membrane remain relatively unchanged (Figure 4).

**Figure 5:**
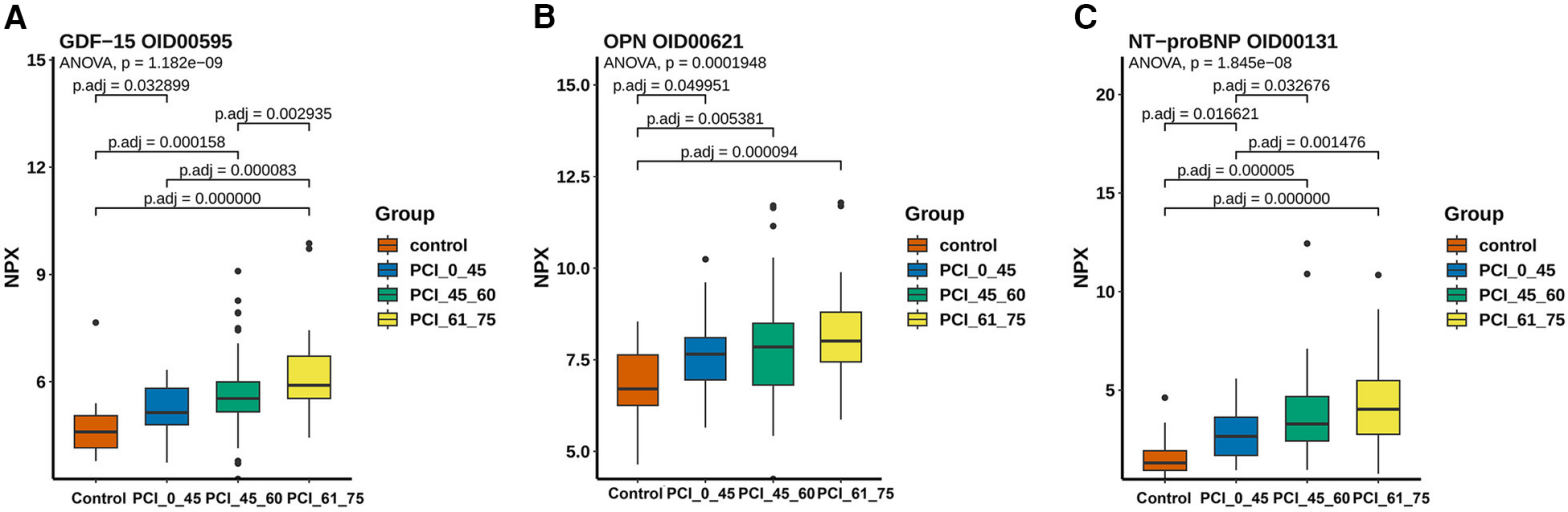
The expression levels of ACS-related proteomics and arteriosclerosis markers. (**A**) The expression of GDF-15 was highest in the 61–75-year-old group and lowest in the control group, with no significant difference between the <45-year-old group and the 45–60-year-old group. (**B**) The lowest expression level was observed in the control group in terms of the content of OPN, and a pattern of increasing expression with age was shown, although no significant difference was shown among the other three groups. (**C**) A significant difference was found between young patients and elderly people aged >45 years based on the expression level of NT-proBNP, with the lowest expression occurring in the control group.

Furthermore, in order to study the diagnostic role of blood protein expression in ACS, we compared the differential protein expression between the control group and different age groups of ACS patients. The results showed that the protein expression of GDF-15 (Figure 5A), osteopontin (OPN) (Figure 5B), and N-terminal pro-brain natriuretic peptide (NT-proBNP) (Figure 5C) was higher in ACS groups of different ages compared to the control group (*p* < 0.05). However, there was no statistical difference in GDF-15 in the age groups under 45 years old and 45–60 years old. OPN showed no statistical difference in different age groups. NT-proBNP showed no statistical difference in the age groups of 45–60 years old and 60–75 years old.

## Discussion

ACS in patients under the age of 45 poses a significant risk to the health and well-being of young individuals, making it crucial to understand their clinical characteristics for effective management and treatment. In this study, we discovered that clinical characteristics and blood proteins expression were associated with a significantly difference in patients with ACS aged younger than 45 years. These factors differed from the traditional cardiovascular risk factors that were more relevant in patients older than 45 years. These findings may have positive implications for diagnosis and management strategies for young patients with ACS.

Previous research has focused on evaluating the influence of standard cardiovascular risk factors on the incidence of CVD, including ACS, in the general population (19, 20). Most studies have reported that ACS primarily affects middle-aged and elderly individuals based on past epidemiological statistics (21). These studies have identified multiple factors, such as hypertension, diabetes mellitus, hypercholesterolemia, smoking habits, and alcohol intake, that are associated with an increased risk of disease (22, 23). However, However, for ACS patients under the age of 45, their risk factors may differ from those traditionally studied. Our follow-up study revealed that age, hypertension, smoking habits, and potential vascular disease may not be the primary pathogenic factors in young patients with ACS, given their age advantages and fewer underlying diseases. Considering the higher BMI observed in young ACS patients, it is reasonable to suspect that the presence of ACS in young adults may be related to obesity and hyperlipidemia. The baseline characteristics confirmed this finding in our study, which revealed several significant differences between the <45-year-old group and the other age groups in terms of hyperlipidemia incidence and BMI.A previous study suggested the need for routine estimation of TC in the diagnosis of CAD in young individuals, which helps in the early detection of myocardial damage and timely intervention, leading to decreased morbidity and mortality (24). In our study, comparisons with other age groups also supported this hypothesis, as the <45-year-old group had the highest concentrations of total cholesterol (TC) and total cholesterol (TAG). Therefore, we speculated that the main factors contributing to early-onset ACS in individuals younger than 45 were BMI and hyperlipidemia. Additionally, a family history of CAD and smoking history have been proven to play key roles in ACS morbidity. Nevertheless, in our study, the prevalence of a family history of CAD among young patients with ACS was also high (32.4%), but this difference was not significant compared to that in the other groups. Similarly, smoking history was found to be a significant factor among the various pathogenic factors in young patients compared to the control group. We speculated that this was due to insufficient sample size. Noninvasive cardiac imaging is widely used to evaluate the presence and prognosis of coronary artery disease (25). Recently, with improvements in imaging technology, noninvasive imaging methods, such as coronary computed tomography angiograms and ultrasonic cardiograms, have also been used for evaluating cardiac function and assessing the presence, severity, and prognosis of coronary artery disease (26, 27). In our study, we observed that, compared with that in young people without ACS, the LVEF in all ACS patients was significantly lower, which indicated decreased cardiac function. However, additional data are needed to determine the differences in cardiac function and prognosis between young patients and older people with ACS in terms of cardiovascular aging.

In addition to these clinical characteristics, we also conducted proteomic analysis using peripheral blood samples from patients to analyze the differences between ACS patients in different age groups and the control population without ACS. The results showed that GDF-15, OPN, and NT-proBNP exhibited significant differences between the control group and ACS patients in different age groups, suggesting that they could serve as diagnostic markers for ACS patients in different age groups. First, GDF-15, a stress-responsive member of the transforming growth factor β cytokine superfamily, has been proven to be an independent and vital predictor of disease development and mortality in patients with ACS (28). GDF-15 is emerging as a prognostic factor in ACS patients and is associated with NSTEMI, unstable angina pectoris, and STEMI, which are the causes of the erosion or rupture of vulnerable atherosclerotic plaques resulting in AMI or death (29–31). According to the investigations by the GUSTO-IV trial, GDF-15 expression is strongly related to all-cause mortality in patients with non-ST-segment-elevation ACS (32). Meanwhile, the 1-year cumulative mortality rates of patients with low, moderately elevated, and significantly elevated GDF-15 concentrations were 1.5%, 5.0%, and 14.1%, respectively. Our study, which confirmed that GDF-15 was expressed at higher levels in ACS patients than in people without ACS, also verified this finding and might indicate potentially different prognoses and mortality rates among the three age groups. A lower concentration of GDF-15 in young patients could indicate a positive signal, ultimately reducing the occurrence of death after ACS. Moreover, the baseline characteristics revealed that patients under 45 years old experienced less severe myocardial damage according to the left ventricular ejection function and the concentrations of vital markers, such as myoglobin and the creatine kinase-myocardial band in the blood, although these differences were not significant. Proteomic analysis provided evidence supporting this observation, as the level of NT-proBNP, which is considered a key marker for reflecting heart failure, was lower in the blood samples of young patients than in those of patients in the other age groups (33–35). Furthermore, OPN, a glycoprotein secreted by macrophages, endothelial cells, and vascular smooth muscle cells, has been identified as a key factor in acute coronary syndrome (ACS) and can be detected in calcified atheromatous plaques, the neointima of injured vessels, and macrophages at sites of inflammation (36–38). Our study also revealed the overexpression of OPN in ACS patients, which confirmed the above perspective. However, no significant differences were not detected between ACS groups of different ages in our study, possibly due to the limited sample size. However, the sample size and follow-up time need to be increased to explore and predict the relationships among the groups in terms of OPN expression. Additionally, it is worth noting that in proteomic analysis, we conducted subcellular localization analysis of proteins. Proteins in cells are purposefully located in their appropriate positions to function. This localization process is a rather delicate process, and under accurate localization conditions, proteins can interact correctly with other biomolecules. Accurately understanding the subcellular localization of proteins is of great significance for revealing biological processes and disease mechanisms. In this study, we found that protein expression in young patients was mostly localized in the extracellular matrix (up to 71.43%), which was significantly different from the other two age groups (60%; 52.25%). This suggests that the activation of extracellular matrix anchoring proteins and protein-protein interactions are significant influencing factors in the pathogenesis of young patients, which can help us accurately predict and further analyze the key biomarkers and mechanisms that affect the disease in young people.

We observed that in ACS patients, as age increases, cytoplasmic proteins gradually increase, while proteins from extracellular sources decrease, while plasma membrane protein levels remain relatively stable. The gradual increase of cytoplasmic proteins may indicate that upregulation of intracellular protein synthesis is a compensatory mechanism for maintaining cellular function and integrity during human aging. The decrease in proteins from extracellular sources indicates a possible decrease in the ability of cells to internalize or utilize external proteins. This reduction may affect the cell's ability to respond to external signals. Plasma membrane proteins play a crucial role in maintaining the basic structure and functional integrity of cells. Its stability ensures appropriate intercellular communication, nutrient transport, and signal transduction. The proteomic differences observed in ACS patients of different age groups may reflect the complex cellular adaptation of the human body to maintain cellular function and integrity in the context of aging and disease. These changes may be related to the different clinical outcomes of ACS patients of different ages. Future research should focus on elucidating the molecular mechanisms of these protein changes and their functional consequences in the context of ACS.

This study has several limitations that remain to be improved upon. First, there was a lack of important information in the collection of patient medical history, such as diet and exercise habits, which directly affect BMI and blood lipid levels (39). Secondly, collecting patient medication usage and treatment information during the experiment would increase the completeness of the study. Thirdly, whether the established heart failure related-proteins such as NT-proBNP, emerging ACS-related biomarkers such as GDF-15, OPN, or other uncovered novel proteins, the different expression patterns in young ACS patients may become a new breakthrough for predicting and managing early-onset ACS in the future. Meanwhile, additional information from patient imaging (angiography) evaluation could help with disease assessment and risk factor prediction. In future researches, the collection and evaluation of patient imaging information will be included. Finally, sufficient data and long-term follow-up are necessary to help us better analyze risk factors and develop diagnostic and treatment plans.

## Conclusion

In summary, compared with other risk factors of ACS, such as old age, hypertension, diabetes, smoking, etc., young ACS patients under 45 years old are more commonly characterized by obesity and hyperlipidemia(including increased total cholesterol and triglycerides). GDF-15, OPN, and NT-proBNP may serve as potential diagnostic markers for young ACS patients. However, further research is needed to confirm the relevance of these factors and to elucidate the potential mechanisms involved. Additionally, this study lacks control groups from different ages other than young patients under 45 years old. These findings possibly may have implications for future management strategies of this specific population.

## Data Availability

The original contributions presented in the study are included in the article/Supplementary Material, further inquiries can be directed to the corresponding authors.

## Supplementary material

Supplementary Figure S1 The distribution of normalized protein expression in all samples. Red: Warning samples (QC failed samples); blue: passed samples (QC passed samples).
