# Supplementary figures and images for: Clinical characteristics and biomarkers feature analysis using a proteomics platform in young patients with acute coronary syndrome
Source: Front Cardiovasc Med. 2024 Aug 13;11:1384546. doi: 10.3389/fcvm.2024.1384546 (PMC11347339; doi:10.3389/fcvm.2024.1384546)

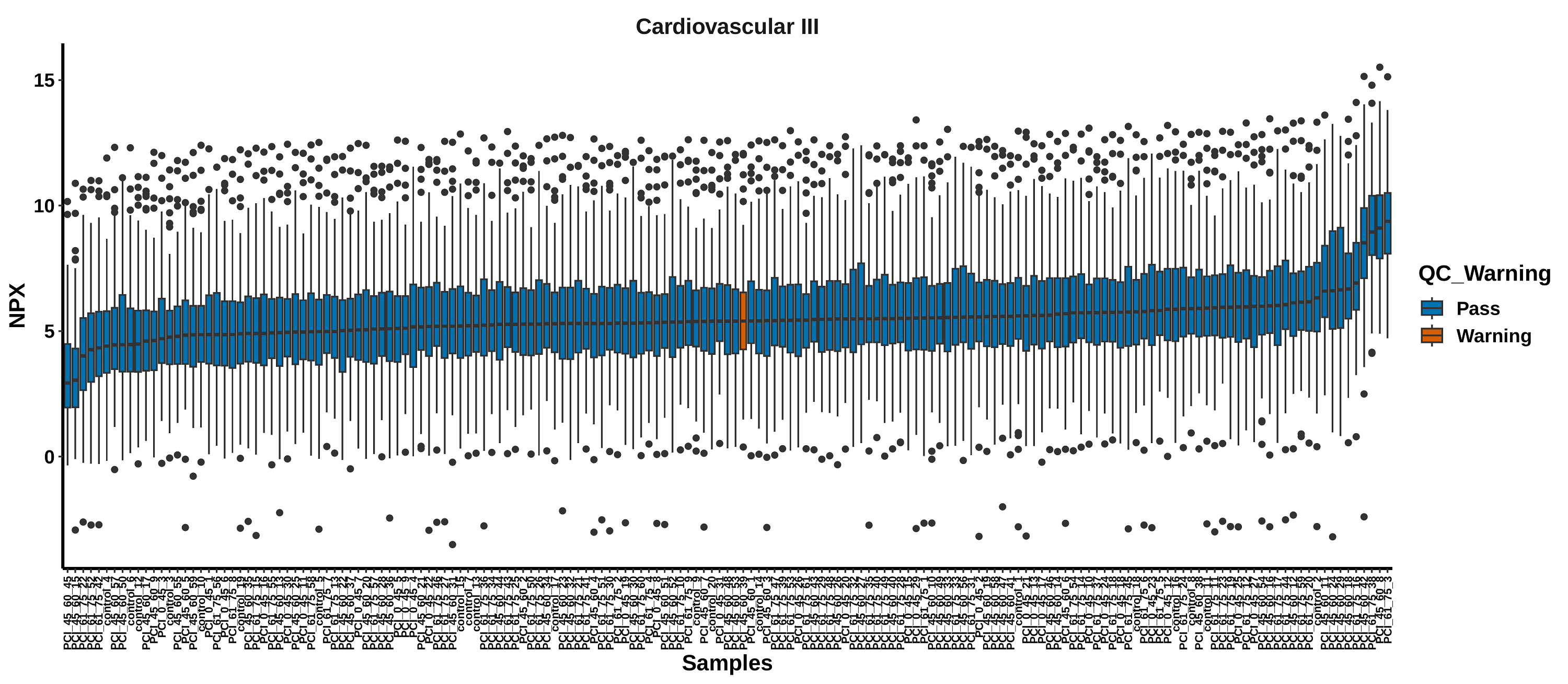

Supplement: Supplementary Figure S1 — The distribution of normalized protein expression in all samples. Red: Warning samples (QC failed samples); blue: passed samples (QC passed samples). [file Image1.tif]
